# Optimization of a Green Ultrasound-Assisted Extraction of Different Polyphenols from *Pistacia lentiscus* L. Leaves Using a Response Surface Methodology

**DOI:** 10.3390/plants9111482

**Published:** 2020-11-03

**Authors:** Cassandra Detti, Luana Beatriz dos Santos Nascimento, Cecilia Brunetti, Francesco Ferrini, Antonella Gori

**Affiliations:** 1 Department of Agriculture, Food, Environment and Forestry (DAGRI), University of Florence, Sesto Fiorentino, 50019 Florence, Italy; cassandra.detti@unifi.it (C.D.); cecilia.brunetti@ipsp.cnr.it (C.B.); francesco.ferrini@unifi.it (F.F.); 2 National Research Council of Italy, Institute for Sustainable Plant Protection (IPSP), Sesto Fiorentino, 50019 Florence, Italy

**Keywords:** Anacardiaceae, design of experiments (DOEs), flavonoids, green extraction, HPLC-DAD, LC-MS/MS, tannins, ultrasound assisted-extraction (UAE)

## Abstract

*Pistacia lentiscus* leaves are used in several applications, thanks to their polyphenolic abundance. Thiswork aimed to characterize the polyphenols and to optimize the extraction conditions to shorten the time, decrease the consumption of solvent, and to maximize the yield of different classes of phenolics, which have diverse industrial applications. The variables were optimized by applying a Box–Behnken design. Galloyl and myricetin derivatives were the most abundant compounds, and two new tetragalloyl derivatives were identified by LC-MS/MS. According to the models, the maximum yields of polyphenols (51.3 ± 1.8 mg g^−1^ DW) and tannins (40.2 ± 1.4 mg g^−1^ DW) were obtained using 0.12 L g^−1^ of 40% ethanol at 50 °C. The highest content of flavonoids (10.2 ± 0.8 mg g^−1^ DW) was obtained using 0.13 L g^−1^ of 50% ethanol at 50 °C, while 0.1 L g^−1^ of 30% ethanol at 30 °C resulted in higher amounts of myricitrin (2.6 ± 0.19 mg g^−1^ DW). Our optimized extraction decreased the ethanolic fraction by 25% and halved the time compared to other methods. These conditions can be applied differently to obtain *P. lentiscus* extracts richer in tannins or flavonoids, which might be employed for various purposes.

## 1. Introduction

*Pistacia lentiscus* L. (Anacardiaceae), known as mastic orlentisk, is an evergreen shrub, widespread over many areas in the Mediterranean basin [1]. This species is largely distributed in dry ecosystems, characterized by nutrient and water scarcity due to the long periods of drought, high irradiation, and temperatures [2,3].

Several studies have demonstrated that *P. lentiscus* leaves are rich in polyphenolic compounds [4,5] including gallotannins and flavonoids (mainly quercetin and myricetin derivatives) [6,7,8,9]. These two main classes of compounds have different industrial and commercial applications. Flavonoids are intensively used in the food industry as preservatives and flavoring agents [10], the cosmetic industry as skin protectors [11], and in agricultureas an anti-infective agents [12]. Tannins, otherwise, are widely applied in the leather industry, as well as beverages additives, corrosion inhibitors of metals in shipbuilding, wood adhesives, and foams [13].

From a pharmacological point of view, both classes of phenolics have long been suggested to have high antioxidant capacities and other several biological activities [14]. *Pistacia lentiscus* leaves have traditionally been used in folk medicine for the treatment of various diseases such as hypertension, stomach aches, and kidney stones [15,16,17,18]. Moreover, anti-ulcer, anti-inflammatory, cytoprotective, acetylcholinesterase inhibition, and anticancer activities have been already described for its leaf extracts [15,19,20,21].

Therefore, leaves of *P. lentiscus* represent a reliable source of polyphenols to be exploited by several industries [22]. Thus, obtaining extracts enriched in different classes of these compounds is of high interest.

The quality and the content of polyphenols in plant leaf extracts depend on several factors such as the harvest moment and seasonality, the plant phenological stage, the leaf age, and the applied extraction process [6,23,24,25]. Well-established conventional extraction methodologies have been associated with significant economic and environmental impacts such as high solvent consumption and prolonged extraction times [23,26]. Nowadays, with the development of the concept of “green extraction”, environmentally friendly techniques should be developed, avoiding hazardous reagents and optimizing extraction parameters such as time, temperature, and solvent type [27,28]. These green techniques, include ultrasound-assisted extraction (UAE), enabling the maximum yield of active compounds with low energy and less time consumed [29,30,31]. Ultrasound-assisted extraction represents one of the best and cheapest technologies with limited instrumental requirements [32,33], being efficiently used for extracting phenolic compounds from several plant materials [23,34,35].

The increasing interest inthe improvement of extraction processes from plants has triggered the application of mathematical models for the optimization of extraction conditions. In this sense, response surface methodology (RSM), widely applied for industrial purposes, has become the most preferable approach for optimizing extraction procedures that apply multiple variables at the same time [36,37]. In this sense, the Box–Behnken design (BBD) is one of the most used RSMs. This design requires a small number of runs and, therefore, avoids time-consuming experiments and has been largely applied for optimizing extractions of single or classes of molecules from different plant materials [38,39,40].

Response surface methodologieshave already been applied for optimizing the ultrasound-assisted extraction of polyphenols from *P. lentiscus* leaves [41]. In this study, the authors tested the UAE by using a central composite design with high solvent volume and leaf material [41]. In addition, the authors quantified the total phenolic content using the Folin–Ciocalteu reagent, not considered specific for phenolics, since this reagent can be reduced by other compounds that might cause interferences in the results [42]. As such, a detailed optimization of different classes of polyphenols in *P. lentiscus* leaf extracts has not yet been conducted.

In this context, this study aimed to:

- Evaluate the effect of different variables (solvent ratio, temperature, extraction time, and ethanol volume) on the UAE of *P. lentiscus* leaves using a first-step screening design;
- Optimize the extraction process, using a Box–Behnken design, in order to obtain extracts with higher amounts of different classes of polyphenols (quantified by high performance liquid chromatography coupled to diode array detection, HPLC-DAD) and applying a greener method than those conventionally used for the extraction of leaves of the species;
- Characterize the major compounds present in the extract with the highest content in polyphenols using liquid chromatography-mass spectrometry (LC-MS/MS).

## 2. Results and Discussions

### 2.1. Screening Design and Determination of the Important Factors

Several factors can influence the efficiency of an extraction such as solvent type, time, particle size, and temperature [43]. Consequently, it is important to verify how different variables affect the extraction of target compounds [23,44]. The UAE is one of the most appropriate extraction processes due to the fact of its efficacy, cleanliness, facility of use, and speed [45,46]. Among the different factors that should be considered in ultrasound-assisted extraction, the polarity of the solvent and the solvent ratio is very important [45,47]. Moreover, time and temperature also affect the yield of the compounds and the costs of the whole process. Indeed, it is desirable to develop methods that lead to a higher extraction of target compounds using lower temperatures, shorter time, and lower concentration of organic solvent than possible [43,47].

The calculated coefficients for the different answers (i.e., total tannins content (TTC), total flavonoids content (TFC), and total polyphenolic content (TPC)) are shown in Table 1. The solvent ratio (x_3_) showed to be the most important factor affecting positively all the responses as can be inferred by the positive and significant value of the b_3_ coefficient. As such, an increase in the solvent ratio from 0.06 to 0.1 L g^−1^ led to higher amounts of polyphenols. Therefore, even higher solvent ratios (solvent volumes) were chosen for the further optimization steps.

The fraction of ethanol (%) (x_4_) showed to be a determinant for the extraction of tannins (TTC) and total polyphenols (TPC), negatively affecting both. The b_4_ coefficient was significant and negative, indicating that the extractions conducted using a smallerpercentage of ethanol resulted in higher yields of these compounds. This phenomenon could be explained by the fact that with an increase in ethanol concentration, the solvent polarity may decrease as well as the molecular movements, reducing the solubility of the polar compounds [48]. In addition, by raising the surface tension of the solvent, an increase in the molecular interactions is induced, consequently raising the extraction [32], while the addition of water to the organic solvent may help break the hydrogen bonding and facilitate the extraction of polyphenols [49]. Based on these results, lower percentages of ethanol were studied in the optimization step in order to conduct a greener extraction [32,50]. In fact, ethanol and water are solvents widely used by food and pharmaceutical industries due to the fact of their safer handling [51].

For the temperature (x_1_), the calculated coefficients (b_1_) were also high for TTC and TPC. For both, this factor showed a positive effect. According to this, higher temperatures (30, 40, and 50 °C) were evaluated during the optimization, which should also be considered when higher amounts of water are applied. The use of higher temperatures during the UAE can increase the efficiency of the extraction process due to the increase in the number of cavitation bubbles formed [45,52]. Moreover, temperature influences the mass transfer process by improving the solvent penetration in plant cells due to the reduction in its viscosity. In addition, higher temperatures increase the degradation of the plant matrix, and weaken the interactions of the polyphenols with other cell constituents, making their extraction easier [49,53].

The coefficient b_2_ showed to be low for all the answers, indicating that the variable x_2_ (time) has no effect on the answers. Therefore, this factor was kept constant in the optimization step, and the shorter extraction time tested (15 min) was chosen. For further industrial purposes, this is desirable, since less time can reduce the energy consumed [54].

### 2.2. Optimization Design: Models and Response Surfaces Analysis

For an efficient extraction process, not only the method used (e.g., UAE, microwave assisted or conventional extractions), but also the variables applied are of a great importance as well as their linear, quadratic, and interactive effects. The multi-factorial study of them, such as applying experimental designs and RSM, allows the maximization of responses with minimal energy loss and solvent consumption [47].

The results of tannins (TTC), flavonoids (TFC), myricitrin (MYC), and total polyphenols (TPC) (calculated as the sum of individual phenolics, Supplementary Materials Tables S1 and S2) obtained for the experimental trials conducted are presented in Table 2 and were used to obtain regression equations (models, Supplementary Materials Table S3). Trials 9 to 11 were shownto have the best conditions for achieving higher amounts of TPC and TTC (Table 2), all of them, interestingly using 40% ethanol as solvent. For the flavonoids content (TFC and MYC) instead, the best trials were from trials 5 to 8, all using 0.15 L g^−1^ of solvent ratio (Table 2). It is interesting to note that for all the responses, an extraction conducted using a solvent ratio of 0.2 L g^−1^ at 40 °C resulted in the lowest amounts of polyphenols (Table 2).

According to the results, second-order polynomial regression models based on the coded coefficient values were obtained for each response (Supplementary Materials Table S3). To verify the fitting of the mathematical models, the data were statistically analyzed. The quadratic model applied is usually assumed to fit the data sufficiently well to indicate the more suitable and the better regions of work. Statistically, the quality of a model is evaluated by the significance of the regression according to the ANOVA test; the lack of fit (LOF), used to measure the adequacy of these models [55]; the multiple determination coefficient (R^2^), which represents the variation of the response explained by the model; and the adjusted multiple determination coefficient (R^2^_adj_), which indicates the capacity of the model to be predictive [55,56,57]. The analysis of the models obtained for the different responses (TTC, TFC, MYC, and TPC) are presented in Table 3.

For a good fit, R^2^ should be at least 80% [58]. In all our analysis, the R² values were higher than 0.89 (89%, Table 3), suggesting that the models described well the behavior of these responses. Moreover, for all of them, the R^2^_adj_ were higher than 0.71, indicating a good predictive power, since in a good statistical model R^2^_adj_ should be comparable and similar to R^2^, with differences less than 0.2–0.3. Furthermore, the values for the LOF were not significant to an extent with the pure error (*p*>0.05, for all). A model will fit the experimental data when a significant regression and a non-significant LOF are found [59]. Therefore, considering these results, as well as the *p*-value (all *p*≤0.05) (Table 3), the models showed to be suitable and appropriate to well describe the relationship betweenthe responses (TTC, TFC, MYC, and TPC) and the independent variables (x_1_ to x_3_).

The significance of the coefficients was also determined (Supplementary Materials Table S3). For the response of total polyphenols (TPC) and total tannin (TTC) contents, the coefficient b_2_ (x_2_–solvent ratio) showed to be the highest, compared with the coefficients b_1_ (x_1_–ethanol fraction, %) and b_3_ (x_3_–temperature). Besides, b_2_ was negative, suggesting that the use of less solvent is better for the extraction of TPC and TTC. Similarly, for the total content of flavonoids (TFC), the coefficient b_2_ showed higher values compared to b_1_ and b_3_, also negatively affecting the response. It means that less solvent should be better for the extraction of flavonoids (Supplementary Materials Table S3). The analysis of variance (ANOVA) for the polynomial models indicated that the quadratic terms of the variable x_1_ (ethanol%, b_11_) and x_3_ (temperature, b_33_) were the most important, significantly influencing the responses (*p* < 0.05) (Supplementary Materials Table S3). In fact, temperature and type of solvent are important factors to be considered in UAE [32].

**Figure 1:**
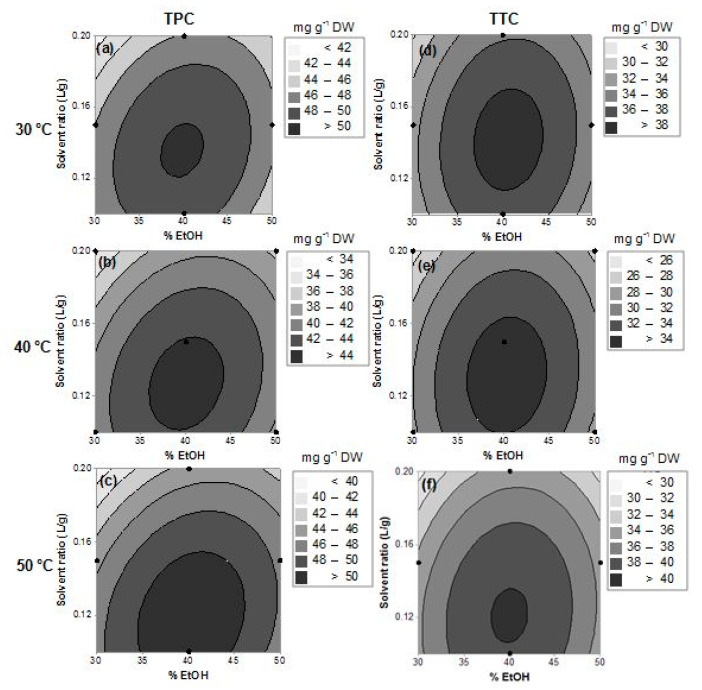
Response surface (contour plots) for predicting the TPC (**a**–**c**) and TTC (**d**–**f**) in *Pistacia lentiscus* leaf extracts with regard to the ethanol fraction (%, x_1_) and solvent ratio (L g^−1^, x_2_), at each temperature (x_3_, 30, 40, and 50 °C). The regions with the darkest-gray color represent the domains of working conditions assuring the maximum values for the evaluated compounds (total polyphenols and tannins).

**Figure 2:**
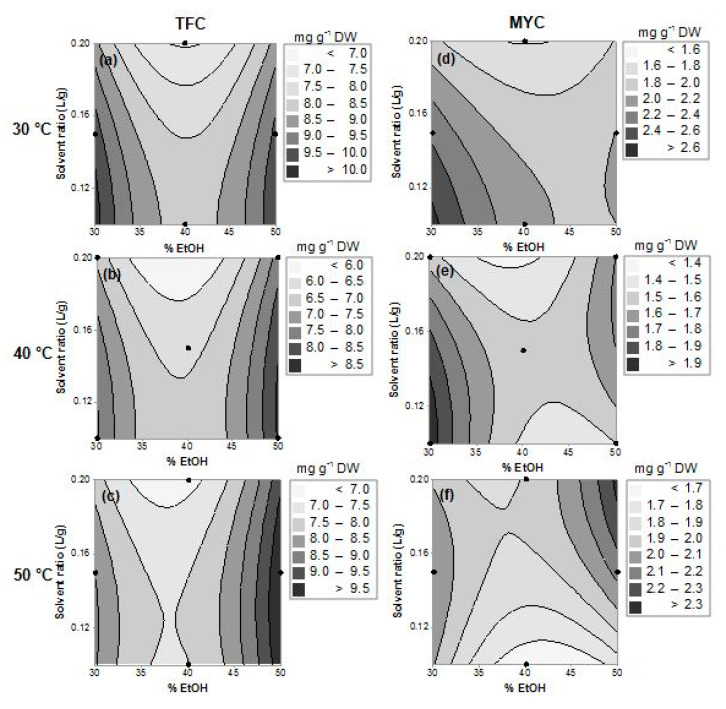
Response surface (contour plots) for predicting the TFC (**a**–**c**) and MYC (**d**–**f**) in *P. lentiscus* leaf extracts with regard to the ethanol fraction (%, x_1_) and solvent ratio (x_2_) used at each temperature (x_3_, 30, 40, and 50 °C). The regions with the darkest-gray or black color represent the domains of working conditions assuring the maximum values for the evaluated compounds (total flavonoids and myricitrin).

To provide a better visualization of the effects of the factors in the responses, contour response surface plots were generated from the models (Figure 1—TTC and TPC and Figure 2—MYC and TFC), by plotting the responses with regard to ethanol concentration (x_1_) and solvent ratio (x_2_) at each temperature 30, 40, and 50 °C (x_3_). These response surfaces can be used for the prediction of the responses (polyphenols contents) in the investigated experimental domain.

For the total polyphenolic content (TPC, Figure 1a–c), 30 °C and 50 °C predicted maximum amounts (>50.0 mg g^−1^ DW, Figure 1a,c), with 50 °C being better, since a more extended and stable optimal region of extraction wasobtained (Figure 1c). At this temperature, 35% to 45% of ethanol in a solvent ratio of 0.1 to 0.15 L g^−1^ should be used (Figure 1c). At 40 °C, a good region was also found, however, resulting in lower amounts (~44.0 mg g^−1^ DW). In fact, the optimal conditions proposed by the model were 40% ethanol in a ratio of 0.12 L g^−1^ at 50 °C, resulting in 51.3 ± 1.8 mg g^−1^ DW of TPC.

A similar percentage of ethanol was also proposed by a previous study focused on the optimization of the phenolic extraction of *P. lentiscus* leaves using a microwave-assisted method [22]. The authors showed that percentages of ethanol around 30% to 40% significantly raised the total phenolic content, spectrophotometrically quantified by the Folin–Ciocalteu reagent [22]. In addition, a similar effect of ethanol percentage was also reported for the extraction of phenolic compounds from other plant sources such as green tea [60]. Considering temperatures analogous to our findings, 45–50 °C was shown to maximize the extraction of polyphenols in *Pistacia atlantica* leaves [36].

The total tannin content (TTC, Figure 1d–f) showed very similar response surfaces and optimal conditions to TPC. Indeed, *P. lentiscus* leaves are rich in tannins, which represent around 70% of the TPC [6]. Therefore, a similar behavior should be expected. For TTC, temperatures of 30 °C and 50 °C should be also used to reach higher amounts of tannins (~40.0 mg g^−1^ DW). However, different from total polyphenols, a narrow optimal region was observed at 50 °C (Figure 1f). At any temperature, an extraction using around 0.13 L g^−1^ of 40% ethanol provided better results (Figure 1d–f). Indeed, the optimal conditions for maximization of the content of tannins were the same for TPC (0.12 L g^−1^, 40% ethanol, 50 °C), yielding 40.2 ± 1.4 mg g^−1^ DW.

For TFC and MYC (Figure 2), at any temperature, a decrease in the content of these compounds was observed around the medium percentages of ethanol with the optimal regions being obtained when extreme values of ethanol fractions (30% or 50%) and temperatures (30 or 50 °C) are chosen (Figure 2). Therefore, higher contents of flavonoids (~10 mg g^−1^ DW, Figure 2a,c) and myricitrin (>2.5 mg g^−1^ DW, Figure 2d,f) are predicted under these conditions. As such, extractions conducted at 50 °C, using 50% ethanol in 0.1 to 0.17 L g^−1^ result in greater amounts of flavonoids (Figure 2c). Decreasing the temperature to 40 °C, lesser amounts of flavonoids are obtained (~8.5 mg g^−1^ DW) (Figure 2b). The increase in the temperature can cause higher solubility and diffusion coefficients of polyphenols, such as flavonoids, which result in a higher extraction rate [61]. The optimal conditions predicted by the model for maximization of the flavonoid content in *P. lentiscus* leaf extracts are 50% ethanol in 0.13 L g^−1^ at 50 °C, resulting in 10.2 ± 0.8 mg g^−1^ DW.

**Figure 3:**
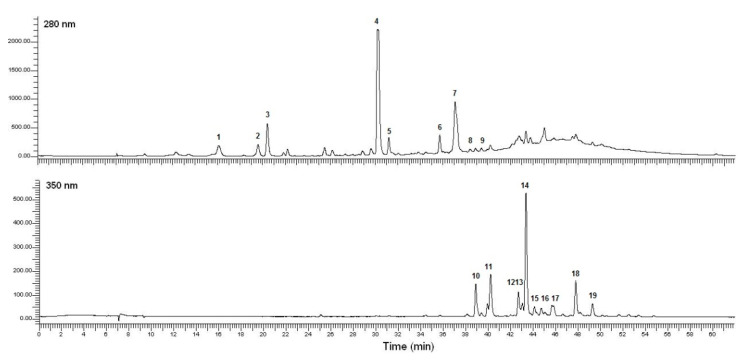
Chromatograms of *P. lentiscus* leaf extracts obtained using the extraction conditions of trial 9 (BBD, Table 2) acquired at 280 nm (above) and 350 nm (below).

Myricitrin content (Figure 2d–f) showed similar response surfaces to the TFC (Figure 2a–c). The extraction conducted at 30 °C with 30% ethanol in 0.1 L g^−1^ should result in maximal contents of this flavonoid (2.6 ± 0.19 mg g^−1^ DW). However, at 50 °C very similar amounts were also obtained, but 50% ethanol in slightly higher volumes should be used (Figure 2f). As can be noticed, the TFC and MYC have similar behaviors. This could be explained because myricitrin is the most abundant flavonoid detected in *P. lentiscus* leaves (Figure 3), also justifying our choice in maximizing its content. This compound has also been described as a major compound in lentisk leaf extracts in previous studies [6,7,62].

To validate the adequacy of the mathematical models, verification experiments were carried out in triplicate under the optimal conditions. Mean values of 39.8 ± 4.1 mg g^−1^ DW for TTC, 50.9 ± 4.9 mg g^−1^ DW for TPC, and 9.9 ± 1.4 mg g^−1^ DW for TFC were obtained from the real experiments and demonstrated the validation of the models for these three responses (*p*_TTC_ = 0.88; *p*_TPC_ = 0.90; *p*_TFC_ = 0.76).

We observed that among the variables tested, the same solvent ratio (~0.13 L g^−1^) and temperature (50 °C) should be used during the UAE process to obtain the maximal yields of tannins and flavonoids. However, the ethanol percentage showed to differ between both classes of compounds. While for tannins (TTC) 40% ethanol should be used, 50% is preferable for the extraction of flavonoids (TFC). This difference can be explained by the distinct solubility of these compounds [47]. The polarity of the ethanol–water mixture decreases with the addition of ethanol, stimulating the extraction of less polar compounds from plant cells. Flavonols, such as myricetin derivatives, show higher solubility with increasing concentration of alcohol, consequently reaching greater extraction yields when less polar solvents are used [23]. Tannins with low molecular weight (galloyl derivatives) occurring in *P. lentiscus* leaf extracts (Figure 3, Table 4) are more polar than the flavonoids detected. Therefore, it is reasonable that the extraction of these types of tannins (and consequently the overall polyphenolic content) is stimulated by the utilization of more polar solvents (i.e., 40% ethanol). Indeed, higher concentrations of ethanol and methanol are more beneficial for the extraction of flavonoids than for tannins, which generally need higher amounts of water [47]. In agreement with our results, Barbouchi et al. [63] obtained higher phenolic contents in *P. lentiscus* leaf extracts when more polar extraction solvents were used. In addition, ethanol was considered the most suitable solvent for the recovery of flavonoids from this species [2].

The extraction conditions optimized here are suitable for experimental and further industrial applications, since they apply a green solvent (ethanol:water) in low quantity (~0.13 L g^−1^) for a short time (15 min), using moderate temperatures (50 °C). Considering that more toxic solvents, such as methanol, chloroform, and ethyl acetate, are intensively used to extract *P. lentiscus* leaves [19,64,65,66] and that the typical extraction methods apply higher percentages of ethanol for longer times [2,6,7], our optimization led to a greener extraction procedure if compared to conventional extraction methods, by decreasing the ethanolic fraction by at least 25% and halved the time used.

A green extraction is defined as a procedure able to reduce energy consumption and use of organic solvents, saving the quality of the process [29]. In particular, three major points should be considered: the improvement and the optimization of the existing methods; the use of simple equipment; and the innovation in the use of alternative solvents [67]. In this sense, the optimization of a standard extraction procedure, especially employing the minimum amount of organic solvent, could be considered green, even if moderate temperatures are applied.

### 2.3. Polyphenolic Composition of the Richest P. lentiscus Extract

Figure 3 shows the polyphenolic profile of *P. lentiscus* leaf extract obtained using the conditions of trial 9 (BBD, Table 2), corresponding to the extract with the highest content of total polyphenols (TPC). The LC-MS/MS analysis was performed to provide a more comprehensive characterization of the polyphenols present in the leaves of the species as well as to confirm previous characterizations reported in the literature [7,9,62,64].

The UV-Vis and MS/MS spectra allowed us to identify 19 compounds (Table 4), classified into three main classes: gallic acid derivatives (peaks 1 and 2), gallotannins (peaks 3–9), and flavonoids (peaks 10–19). For flavonoids, three peaks were identified as myricetin derivatives (peaks 10, 11, 14), six as quercetin derivatives (12, 13, 15–18), and one as a kaempferol derivative (19).

Among the four main peaks detected, three of them (peaks 3, 4, and 7) showed the fragmentation correspondent to mono-, di-, and trigalloylquinic acids, respectively (Table 4). These metabolites have already been described in the literature using different kinds of detectors such as triple quadrupole (QQQ) [7,9,64] and quadrupole time-of-flight (Q-TOF) mass spectrometers [62]. Indeed, according to these previous reports, the monogalloylquinic acid (peak 3) was defined by the fragments *m/z* 343 [M − H]^−^ and 191; this lastresulted from the loss of the galloyl moiety [M152-H]. In addition, the digalloylquinic acid and its isomer (peaks 4 and 5) were characterized by the fragments *m/z* 495 [M − H]^−^, 343, 191, and 169, that are consistent with the successive loss of two galloyl units, and correspondent to the gallic acid itself (*m/z* 169). Finally, the trigalloylquinic acid (peak 7) and its isomer (peak 6) showed the fragments *m/z* 647 [M − H]^−^, 495, 343, 191, and 169, consistent with a trigalloyl substitution. Two minor peaks with the UV spectra and the mass fragmentation typical of quinic acid derivatives were also detected, with precursor ions of *m/z* 799 and fragments 495, 343, 191, and 169, correspondent to four consecutive losses of galloyl moieties (Supplementary Materials Figure S1). As such, these peaks (peaks 8 and 9) were tentatively identified as tetragalloylquinic acid (and its isomer), here, firstly reported in *P. lentiscus* leaf extracts.

Among the gallic acid derivatives (peaks 1 and 2), the peak 1 was assigned as monogalloyl glucose (glucogallin), based on the literature [7] and according to its ion fragments *m/z* 331 [M − H]^−^, 169 (resulted from the loss of the glucose, *m/z* 162) and 125 (derived from the decarboxylation of galloyl). The peak 2, instead, was identified as gallic acid, based on its characteristic mass spectra, with the precursor ion at *m/z* 169 [M − H]^−^ and the fragment *m/z* 125 (decarboxylation of the galloyl). The identification of this compound was also confirmed based on the comparison with the specific external standard.

Flavonoids (from peak 12 to 18) were identified based on the mass fragment of their corresponding aglycon units, namely, myricetin (*m/z* 317), quercetin (*m/z* 301), and kaempferol (*m/z* 285). This was confirmed by the injection of the external standards myricitrin, rutin, and kampferol-3-*O*-rutinoside. The sugar moieties were characterized based on the neutral losses of 132 (presence of pentosides: xylose or arabinose), 162 (hexosides: galactose or glucose), and 146 (deoxyhexoside: rhamnose). Thus, in agreement with the fragmentation patterns described in the literature [62,64], the following flavonoids were tentatively identified as: myricetin-3-*O*-galactoside (peak 10), myricetin-3-*O*-rutinoside (peak 11), quercetin-*O*-hexosides 1 and 2 (peaks 13 and 15), quercetin-*O*-galloyl-pentoside (peak 16), quercetin-3-*O*-arabinoside (peak 17), quercetin-3-*O*-rhaminoside (peak 18), and kaempferol-*O*-hexoside (peak 19). The identification of the major flavonoidic peak (14), myricetin-3-*O*-rhamnoside (myricitrin), was obtained by comparison with the specific analytical standard. The remaining peak (12) was tentatively identified as a quercetin derivative based on the UV–Vis spectra, in the absence of conclusive mass-spectrometric data and reference in the literature.

High contents of gallotannins (galloylquinic acid derivatives) and myricetin derivatives were previously described in *P. letiscus* leaves [6,7,9,17,68]. These compounds represent approximately 90% of the polyphenolic composition of the leaf extracts [6] and are possibly the main responsible for their biological properties such as: anti-inflammatory, cytoprotective, hepatoprotective, enzymatic-inhibitory, antitumor, and anti-diabetes [7,62,69,70,71]. It is noteworthy that the two molecules here tentatively identified as tetragalloylquinic acid derivatives have not been described yet in lentisk leaves. These compounds have shown to possess high activity against bronchial hyperreactivity and allergic reactions [72].

All these reports show the importance of developing new methodologies in order to increase the content of active compounds in *P. lentiscus* extracts. This could lead to a wider application of the extracts as nutraceuticals, medicines, or as sources of substances for different commercial and industrial applications.

In fact, the compounds detected here showed important biological activities. In particular, galloyl derivatives of quinic acid have been shown to have effective inhibition of Fe_2_^+^-induced lipid peroxidation in cells [73], anti-HIV, anti-allergic [74], and high antioxidant activities [68]. This class of molecules is among the most pharmacologically active natural products detected in several plant species [75]. In addition, gallotannins are applied as wood adhesives in the leather manufacturing as well as in the construction sector [13].

Flavonoids, especially with quercetin and myricetin skeleton, are considered powerful antioxidants distributed in several plant species with proven anti-inflammatory and anti-cancer actions [76,77,78,79]. Besides their application as medicinal compounds, they are specially utilized in cosmetic and nutraceutical products [11].

Moreover, the most abundant flavonoid detected in all extracts, the myricetin-3-*O*-rhamnoside, showed a noticeable lipid peroxidation inhibitionin in vitro tests, with very low IC_50_ (inhibitory concentration at 50%) [80], being even more effective as antioxidant than vitamin C [81]. In addition, this molecule has demonstrated positive effects against the oxidative stress induced by hyperglycemia in C2C12 cells [82] and has shown significant inhibition in peroxynitrite-mediated DNA damage [83].

## 3. Materials and Methods

### 3.1. Plant Material

Fully expanded leaves from branches at the top of the canopy were randomly collected from adult plants of *P. lentiscus* growing in the coastal dunes of Southern Tuscany, Italy (42°46′ N, 10°53′ E). Harvesting was conducted in July 2019, around midday in order to ensure the high polyphenolic composition of the leaves [6].

After collection, the leaves were cleaned (to remove damaged parts, dust, and other contaminants from the natural habitat), immediately frozen in liquid nitrogen, freeze-dried, ground into a fine powder, and kept at −80 °C until the moment of extraction.

### 3.2. Ultrasound-Assisted Extraction (UAE) Procedure

Freeze-dried ground leaves (0.15 gweighted on a digital analytical balance Precisa^®^ 125A) were extracted using ethanol in different percentages and volumes according to the design matrixes (Table 1 and Table 2). The UAE was conducted in an ultrasonic bath (BioClass^®^ CP104) using a constant frequency of 39 kHz and an input power of 100 W. The different temperatures and times, according to each trial (Table 1 and Table 2), were monitored with a thermometer (Weber^®^ 6750, Springfield, Illinois, USA) and a timer (Fisher Scientific^®^, Los Angeles, CA, USA). After the extraction, the samples were centrifuged (5 min, 9000 rpm, 5 °C-ALC^®^ 4239R, Milan, Italy) and the supernatants were partitioned with 3 × 5 mL *n*-hexane, in order to remove lipophilic compounds that could interfere with the analysis. The hydroethanolic phase was reduced to dryness using a rotavapor (BUCHI^®^ P12, Cornaredo, Italy; coupled to a vacuum controller V-855), and the residue was resuspended with 1.0 mL of MeOH: Milli-Q_H20_ solution (1:1 *v*/*v*, pH 2.5 adjusted with HCOOH). These samples were used to conduct the HPLC-DAD analysis for the quantification of the different classes of polyphenols to construct the model. In addition, the extract with the highest polyphenolic content was chosen for the LC-MS/MS analysis in order to furnish a detailed characterization of its chemical composition.

### 3.3. HPLC-DAD Quantification and LC-MS/MS Characterization of the Extracts

High performance liquid chromatography coupled to diode array detection (HPLC-DAD) was used for quantification of the different polyphenolic classes of the extractsobtained at the different conditions tested.

The samples (5 µL) were injected into a Perkin^®^ Elmer Flexar liquid chromatography equipped with a quaternary 200Q/410 pump and an LC 200 diode array detector (DAD) (all from Perkin Elmer^®^, Branford, Connecticut, USA). The stationary phase consisted in a Zorbax^®^ C-18 column (250 mm × 4.6 mm, 5 µm particle size) and the eluents were (A) acidified water (0.1% HCOOH) and (B) acetonitrile (0.1% HCOOH).The following gradient was applied: 1 min (3% B), 1–55 min (3–40% B), 55–60 min (40% B), 60–61 min (3% B), with 62 min of total analysis time, in a flow rate of 0.6 mL min^−1^. Ten minutes of conditioning step were used to return to the initial conditions of the method.

The identification of the polyphenols with HPLC-DAD was carried out based on the retention time, UV-Vis spectral characteristics, comparison with those of the authentic standards acquired at 280 and 350 nm, as well as on the subsequent LC-MS/MS analysis. Quantifications were made by HPLC-DAD. The standards (gallic acid, myricitrin, rutin, and kaempferol-3-*O*-rutinoside from Sigma–Aldrich^®^–Merck^®^KGaA, Darmstadt, Germany) were used to obtain five-point calibration curves. If a commercial standard was not available, quantification was performed using the calibration curve of standards from the same phenolic group. The linearity of these calibration curves was determined by the coefficient of determination (R²), being higher than 0.999 for all the three standards. The limit of detection (LOD) and quantification (LOQ), both expressed as μg/mL, were calculated using signal-to-noise ratio of 3 and 10, respectively [84]. The following limits of detection and quantification were found for the standards: LOD_gallic acid_ = 0.3 and LOQ_gallic acid_ = 0.85; LOD_rutin_ = 0.28 and LOQ_rutin_ = 0.6; LOD_myricitrin_ = 0.12 and LOQ_myricitrin_ = 0.38; LOD_kaempferol-3-O-rutinoside_ = 0.21 and LOQ_kaempferol-3-O-rutinoside_ = 0.49).

All the extracts were analyzed in triplicate. The quantitative results of the polyphenols (reported as mg per g of dry weight, DW) were expressed as: myricitrin (the most abundant flavonoid detected in the *P. lentiscus* leaf extracts), total tannin, total flavonoid, and total polyphenols contents, represented as the sum of individual tannins (TTC), flavonoids (TFC) and polyphenols (TPC) detected by HPLC-DAD analysis in each extract (Supplementary Materials Tables S1 and S2).

The characterization of polyphenols was conducted utilizing a LC–DAD-MS/MS system consisted of a Shimadzu^®^ LCMS-8030 triple quadrupole mass spectrometer (Kyoto, Japan) operated in the electrospray ionization (ESI) negative mode and a Shimadzu^®^Nexera HPLC system (Kyoto, Japan), coupledto a diode array detector (DAD). A reversed-phase Waters^®^ Nova-Pak C18 column (4.9 × 250 mm, 4 μm; Waters^®^, Milford, MA, USA) was used. The mobile phase consisted of water (1% HCOOH, solvent A) and acetonitrile (1% HCOOH, solvent B) and the separation was conducted using the following gradient: 2% B isocratic (10 min), from 2% to 98% B (30 min), 98% B isocratic (7 min) in a flow rate of 0.6 mL min^−1^ and 10 µL of injection volume. The conditions for MS analysis were nitrogen as nebulizing and drying gas (at flow rates of 3.0 and 15.0 L min^−1^, respectively); interface voltage of –3.5 kV; desolvation line temperature of 250 °C; heat block temperature of 400 °C. The spectrometer operated in product ion scan mode using analyte-specific precursor ions; and argon was used as collision-induced dissociation (CID) gas (at 230 kPa). Identification of individual phenolics was carried out by comparison with retention times, UV-Vis, MS and MS/MS spectra, bibliographic data, and available external standards injected in the same conditions (gallic acid, myricitrin, rutin, and kampferol-3-*O*-rutinoside, all from SigmaAldrich^®^–Merck^®^KGaA, Darmstadt, Germany).

### 3.4. Experimental Designs: Optimization Procedure and Data Analysis

The factors affecting the ultrasound-assisted extraction (Section 3.2.) were firstly screened using a fractional factorial design (FFD) (2^4−1^) in order to select the variables and levels to be applied during the optimization step. Based on the results, a Box–Behnken design was conducted to determine the best combination of the important variables selected [56].

#### 3.4.1. Screening Fractional Factorial Design: Selection of the Important Variables for the Extraction Optimization

Four factors: temperature (x_1_; 5 °C or 30 °C), time (x_2_; 15 or 30 min), solvent ratio (x_3_; 0.06 or 0.1 L g^−1^) and ethanol fraction (x_4_; 50% or 75% *v*/*v*) were chosen as independent variables and analyzed in two levels (+1, −1; FFD 2^4−1^; Table 1). The variables and their levels were initially chosen based on their importance for the UAE of plant materials [47]. Nine trials were conducted (8 trials + central point), in different combinations of the variables (x_1_ to x_4_ inTable 1), all in triplicate. The details of the UAE process conducted are described in the Section 3.2.

The main effects of each factor (x_1_ to x_4_) in the following responses: total tannins (TTC), total flavonoids (TFC), and total polyphenolic contents (TPC) were estimated by the calculation of the coefficients of each variable (b_1_, b_2_, b_3_, and b_4_) using the statistical software Minitab^®^ 18 (LCC, Pennsylvania, USA). The factors that were significant in the regression analysis (*p* ≤ 0.05) were considered to have an impact on the responses and selected for the optimization step.

#### 3.4.2. Box–Behnken Design for Optimization of the Extraction Conditions

After the determination of the most important factors, these variables were optimized using a Box–Behnken design, a simple and more efficient three-level factorial design in comparison to other 3^3^ designs [39]. Three independent variables (factors) were analyzed in three levels: temperature (x_1_; 30, 40, and 50 °C), solvent ratio (x_2_; 0.1, 0.15, and 0.2 L g^−1^), and ethanol fraction (x_3_; 30, 40 and 50%, *v*/*v*).

Fifteen experimental trials, resulted from the combination of the three levels (−1, 0, 1) of each variable and three replicates of the central point were thus conducted in triplicate, following the BBD matrix (Table 2). The response variables (i.e., TTC, TFC, MYC, and TPC) were fitted to a second-order polynomial model equation (Equation(1)) that was used to predict the optimum conditions of extraction process and to construct the response surfaces (RSM).

(1) $$Y=\beta 0+\overset{k}{\underset{i=1}{\sum }}\beta_{i}X_{i}+\overset{k}{\underset{i=1}{\sum }}\beta_{ii}X_{i}^{2}+\overset{k}{\underset{i=1}{\sum }}\overset{k-1}{\underset{j=i+1}{\sum }}\beta_{ij}Xij$$

where *Y* represents the response variables, *Χi* and *Χj* are the independent variables affecting the response, *β*0, *βi*, *βii*, and *βij* are the regression coefficients of the model (intercept, linear, quadratic, and interaction terms, respectively), and k is the number of variables (*k* = 3). The variables and their levels, with both coded (−1, 0, 1) and uncoded (real values) are given in Table 2.

The Minitab^®^18 software (LCC, State College, PA, USA) was used for the RSM data analysis. To test the significance of the models, an ANOVA with 95% confidence level was carried out for each response. Furthermore, a lack of fit (LOF) test was performed to check the variability of the residues of the proposed models. The estimated coefficients of multiple determination (R^2^) of the quadratic models and the adjusted coefficients of multiple determination (R^2^_Adj_) were also calculated. These coefficients reflect the fraction of the total variability in the response that is explained by the model.

In order to verify and validate the predicted optimal UAE conditions, experimental extractions were under the conditions selected as optimal for TTC, TPC (0.12 L g^−1^ of 40% ethanol, at 50 °C), and TFC (0.13 L g^−1^ of 50% ethanol, at 50 °C). The predicted and experimental responses were compared by a *t*-test and the model validation was confirmed if *p* > 0.05.

## 4. Conclusions

In conclusion, this study was able to define the optimal UAE conditions to obtain higher amounts of different polyphenolic classes from *P. lentiscus* leaves in a greener way when compared to conventional extraction methods, using a low percentage of organic solvent and less time consumed.

According to our findings, among the variables tested (i.e., temperature, ethanol fraction, and volume), the optimal conditions were slightly different only in terms of ethanol percentage: 40% for tannins and 50% for flavonoids but similar in solvent ratio (~0.13 L g^−1^), temperature (50 °C), and time (15 min). A good agreement between the experimental and the predicted values at these optimal conditions showed the adequacy of the models obtained. Furthermore, this work brings novelty in the characterization of *P. lentiscus* leaf extracts, putatively identifying for the first time the presence of two tetragalloyllquinic acid derivatives.

These results are important considering the wide commercial applications of the different polyphenolic classes of this species, as well as the new trend in the green chemistry. Moreover, they may constitute the basis for future UAE processes applied in larger scale conditions.

## Figures and Tables

**Table 1:** Screening fractional factorial design matrix (FFD-2^4−1^) of trials conducted (trials 1 to 9, this last the central point), with the independent variables (x_1_ to x_4_) and answers (y).

Experimental Trials	Independent Variables (x)							
	x_1_ (Temperature, in °C)		x_2_ (Time, in min)		x_3_ (Solvent Ratio, in L g^−1^)		x_4_ (Ethanol Fraction, in % *v/v*)	
1	5	(−)	15	(−)	0.06	(−)	50	(−)
2	25	(+)	15	(−)	0.06	(−)	75	(+)
3	5	(−)	30	(+)	0.06	(−)	75	(+)
4	25	(+)	30	(+)	0.06	(−)	50	(−)
5	5	(−)	15	(−)	0.1	(+)	75	(+)
6	25	(+)	15	(−)	0.1	(+)	50	(−)
7	5	(−)	30	(+)	0.1	(+)	50	(−)
8	25	(+)	30	(+)	0.1	(+)	75	(+)
9	15	(0)	22.5	(0)	0.08	(0)	62.5	(0)
**Answers (y)**	**Calculated coefficients**							
**TTC**	b_1_	0.338	b_2_	−0.104	b_3_	0.571 *	b_4_	−0.604 *
**TFC**	b_1_	−0.092	b_2_	−0.058	b_3_	0.258 *	b_4_	0.008
**TPC**	b_1_	0.375	b_2_	−0.108	b_3_	1.267 *	b_4_	−0.700 *

The trials were conducted in triplicate. The coefficients (b_1_ to b_4_), correspondent to each variable (x_1_ to x_4_), were calculated by regression. The asterisks (*) indicate significant coefficients (*p* ≤ 0.05).Total tannins content (TTC); total flavonoids content (TFC); total polyphenolic content (TPC).

**Table 2:** Box–Behnken design (BBD) matrix with natural and coded values for the independent variables and responses. Fifteen experimental trials were conducted with triplicates of the central point (trial 13).

	Independent Variables (Factors)						Dependent Variables (Responses)			
Trials	x_1_ (Ethanol Fraction, in % *v/v*)		x_2_ (Solvent Ratio, in L g^−1^)		x_3_ (Temperature, in °C)		TTC	TFC	MYC	TPC
1	30	(−)	0.1	(−)	40	(0)	30.5	8.6	2.0	42.6
2	50	(+)	0.1	(−)	40	(0)	30.1	8.9	1.4	39.9
3	30	(−)	0.2	(+)	40	(0)	24.3	6.5	1.6	33.2
4	50	(+)	0.2	(+)	40	(0)	26.5	7.1	1.6	35.6
5	30	(−)	0.15	(0)	30	(−)	31.9	9.5	2.2	44.0
6	50	(+)	0.15	(0)	30	(−)	33.6	9.6	2.1	46.0
7	30	(−)	0.15	(0)	50	(+)	35.7	8.5	1.9	45.9
8	50	(+)	0.15	(0)	50	(+)	36.6	10.1	2.2	49.4
9	40	(0)	0.1	(−)	30	(−)	37.6	8.0	2.0	49.0
10	40	(0)	0.2	(+)	30	(-)	37.8	7.5	1.6	48.6
11	40	(0)	0.1	(−)	50	(+)	37.7	6.9	1.6	48.6
12	40	(0)	0.2	(+)	50	(+)	34.5	7.1	1.9	44.6
13	40	(0)	0.15	(0)	40	(0)	34.9 ± 1.3	5.8 ± 0.60	1.4 ± 0.15	44.3 ± 1.7

For the central point (trial 13), values of the mean ± SD are presented. All the values of the responses are expressed in mg g^−1^ DW. Total tannins content (TTC); total flavonoids content (TFC); myricitrin content (MYC), total polyphenolic content (TPC).

**Table 3:** Statistical parameters after data analysis and fit of the models obtained for the different responses.

Responses	Analysis of the Model				Lack of Fit (LOF)	
	R^2^	R^2^_adj_	F-Value of Model	*p*-Value of Model	F-Value of Lack of Fit	*p*-Value of Lack of Fit
**TTC**	0.95	0.74	5.32	0.040 *	3.56	0.23
**TFC**	0.90	0.73	5.13	0.043 *	1.52	0.42
**MYC**	0.89	0.71	4.79	0.049 *	1.25	0.47
**TPC**	0.90	0.71	4.80	0.049 *	3.07	0.26

* Significant values (*p* ≤ 0.05).

**Table 4:** LC–DAD-MS/MS characterization of the main polyphenols present in extracts of *P. lentiscus* leaves. Compounds numbers correspond to those indicated in Figure 3 (sh, shoulder).

Peak	t_R_, (min)	λ _max_, (nm)	Collision Energy, (V)	[M − H]^−^,(*m/z*)	MS^2^, (*m/z*)	Peak Assignment
1	16.23	234,270	10	331	169,151,125	Monogalloyl glucose
2	19.71	234,272	10	169	125	Gallic acid
3	20.53	236,272	15	343	191	Monogalloyl quinic acid
4	30.24	236,276	15	495	343,191,169	Digalloyl quinic acid (isomer 1)
5	31.22	236,276	15	495	343,191,169	Digalloyl quinic acid (isomer 2)
6	35.72	256,356	20	647	495,343,191,169	Trigalloyl quinic acid (isomer 1)
7	37.09	256,356	20	647	343,191,169	Trigalloyl quinic acid (isomer 2)
8	38.47	265,355	20	799	495,343,191,169	Tetragalloyl quinic acid (isomer 1)
9	38.59	265,355	20	799	495,191,169	Tetragalloyl quinic acid (isomer 2)
10	38.83	264,314,346 sh	10	479	317,316	Myricetin-3-*O*-galactoside
11	40.15	268,314,348sh	15	625	479,316,317	Myricetin-3-*O*-rutinoside
12	42.62	256,350	10	493	301	Quercetin derivative
13	42.94	256,350	10	463	381,300,301	Quercetin-*O*-hexoside 1
14	43.29	260,358,346 sh	10	463	316,271,179	Myricitrin (Myricetin-3-*O*-rhamnoside)
15	44.23	270,350,300 sh	10	463	381,300,301	Quercetin-*O*-hexoside 2
16	44.97	256,350	15	585	525,301,179	Quercetin-*O*-galloyl-pentoside
17	45.57	256,350,300 sh	10	433	300,301	Quercetin-3-*O*-arabinoside
18	47.69	266,350,300 sh	15	447	300,301	Quercitrin (Quercetin-3-*O*-rhamnoside)
19	49.19	265,348	15	447	415,365,285	Kaempferol-*O*-hexoside

## Supplementary Materials

The following are available online at https://www.mdpi.com/2223-7747/9/11/1482/s1, Table S1:Polyphenolic content (in mg g^−1^ DW) of individual tannins and gallic acid derivatives obtained in each trial of the optimization BBD design; Table S2: Flavonoidic content (in mg g^−1^ DW) of individual compounds obtained in each trial of the optimization BBD design; Table S3: Regression coefficients (intercept, linear, quadratic, and interaction) of the models obtained for each response (total tannin—TTC, total flavonoids—TFC, myricitrin—MYC, and total polyphenols—TPC contents); Figure S1. MS/MS spectra of tetragalloyl quinic acids (isomer 1, A and isomer 2, B) corresponding to peak 8 and 9 of Table 4, respectively
