# Supplementary material for: Optimization of a Green Ultrasound-Assisted Extraction of Different Polyphenols from Pistacia lentiscus L. Leaves Using a Response Surface Methodology
Source: Plants (Basel). 2020 Nov 3;9(11):1482. doi: 10.3390/plants9111482 (PMC7692629; doi:10.3390/plants9111482)
Supplement: Supplementary file 1 [file plants-09-01482-s001.pdf]

Table S1. Content of individual tannins and gallic acid derivatives (mg g^{-1} of DW) corresponding to peaks 1–9 reported in Figure 3 and Table 4 obtained in each trial of the optimization BBD design (Table 2). MG-Glu – monogalloyl glucose; GA – gallic acid; MQA – monogalloyl quinic acid; DQA (digalloyl quinic acid); TQA (trigalloyl quinic acid); TTQA (tetragalloyl quinic acid); TTC – total tannin content; TPC – total polyphenols content*.

Trials	x_1	x_2	x_3	Peak 1 (MG-glu)	Peak 2 (GA)	Peak 3 (MQA)	Peak 4 (DQA1)	Peak 5 (DQA2)	Peak 6 (TQA1)	Peak 7 (TQA2)	Peak 8 (TTQA1)	Peak 9 (TTQA2)	TTC	TPC
**1**	**30**	**0.1**	**40**	1.3 ± 0.2	2.2 ± 0.3	3.6 ± 0.4	14.9 ± 0.88	1.9 ± 0.2	1.8 ± 0.2	6.7 ± 0.8	0.95 ± 0.09	0.65 ± 0.09	30.5 ± 3.1	42.6 ± 2.8
**2**	**50**	**0.1**	**40**	0.31 ± 0.05	0.60 ± 0.03	4.3 ± 0.46	13.9 ± 0.9	2.5 ± 0.3	1.5 ± 0.3	6.2 ± 0.8	0.93 ± 0.17	0.74 ± 0.14	30.1 ± 1.3	39.9 ± 0.9
**3**	**30**	**0.2**	**40**	0.96 ± 0.01	1.5 ± 0.05	1.7 ± 0.2	15.4 ± 1.1	1.3 ± 0.4	0.91 ± 0.05	4.2 ± 0.5	0.48 ± 0.05	0.36 ± 0.04	24.3 ± 2.4	33.2 ± 0.80
**4**	**50**	**0.2**	**40**	0.66 ± 0.04	1.3 ± 0.07	2.7 ± 0.3	16.0 ± 1.2	1.6 ± 0.2	1.5 ± 0.08	3.7 ± 0.4	0.58 ± 0.06	0.42 ± 0.03	26.5 ± 0.9	35.6 ± 1.7
**5**	**30**	**0.15**	**30**	0.76 ± 0.06	1.8 ± 0.11	3.5 ± 0.4	18.1 ± 2.0	1.8 ± 0.3	2.1 ± 0.4	5.4 ± 0.7	0.63 ± 0.06	0.40 ± 0.05	31.9 ± 2.6	44.0 ± 2.2
**6**	**50**	**0.15**	**30**	1.6 ± 0.09	1.1 ± 0.07	4.5 ± 0.5	15.9 ± 1.2	1.6 ± 0.2	2.3 ± 0.3	7.9 ± 0.8	0.89 ± 0.07	0.51 ± 0.06	33.6 ± 0.4	46.0 ± 1.3
**7**	**30**	**0.15**	**50**	0.50 ± 0.03	1.1 ± 0.02	4.6 ± 0.6	16.5 ± 2.0	2.4 ± 0.3	2.2 ± 0.3	8.7 ± 0.9	0.70 ± 0.10	0.58 ± 0.07	35.7 ± 2.1	45.9 ± 1.2
**8**	**50**	**0.15**	**50**	1.4 ± 0.2	1.3 ± 0.3	4.8 ± 0.5	17.2 ± 1.8	2.1 ± 0.3	2.5 ± 0.4	8.5 ± 0.7	0.89 ± 0.2	0.66 ± 0.5	36.6 ± 1.1	49.4 ± 0.5
**9**	**40**	**0.1**	**30**	1.9 ± 0.4	1.5 ± 0.2	5.4 ± 0.6	17.0 ± 1.5	2.7 ± 0.4	2.5 ± 0.1	8.3 ± 0.9	1.0 ± 0.09	0.73 ± 0.08	37.6 ± 2.4	49.0 ± 2.2
**10**	**40**	**0.2**	**30**	1.7 ± 0.3	1.6 ± 0.4	5.2 ± 0.6	17.8 ± 2.0	2.5 ± 0.3	2.3 ± 0.3	8.3 ± 1.1	0.87 ± 0.08	0.88 ± 0.09	37.8 ± 0.7	48.6 ± 1.2
**11**	**40**	**0.1**	**50**	1.9 ± 0.1	2.1 ± 0.3	5.6 ± 0.7	17.5 ± 1.8	2.2 ± 0.3	2.3 ± 0.5	8.4 ± 0.9	1.0 ± 0.1	0.80 ± 0.2	37.7 ± 2.5	48.6 ± 1.2
**12**	**40**	**0.2**	**50**	1.5 ± 0.2	1.5 ± 0.3	4.2 ± 0.5	15.7 ± 1.3	2.3 ± 0.1	2.6 ± 0.3	7.7 ± 0.8	1.1 ± 0.1	0.90 ± 0.07	34.5 ± 1.5	44.6 ± 1.2
**13**	**40**	**0.15**	**40**	2.1 ± 0.3	1.5 ± 0.4	4.5 ± 0.7	18.7 ± 1.8	1.6 ± 0.3	1.7 ± 0.4	6.2 ± 0.7	1.2 ± 0.2	0.97 ± 0.08	34.9 ± 1.3	44.3 ± 1.7

*TPC – sum of TTC, TFC (Table S2), MG-glu and GA.

Table S2. Content of individual flavonoids (mg g^{-1} of DW) corresponding to peaks 10–19 reported in Figure 3 and Table 4 obtained in each trial of the optimization BBD design (Table 2). MYR-3-*O*-Gal—Myricetin-3-*O*-galactoside; MYR-3-*O*-Rut—Myricetin-3-*O*-rutinoside; QUE der—Quercetin derivative; QUE-*O*-hex1—Quercetin-*O*-hexoside 1; MYC—Myricitrin (Myricetin-3-*O*-rhamnoside); QUE-*O*-hex2—Quercetin-*O*-hexoside 2; Q-*O*-Galpent—Quercetin-*O*-galloyl-pentoside; QUE-3-*O*-Ara—Quercetin-3-*O*-arabinoside; QUE-3-*O*-Rha—Quercitrin (Quercetin-3-*O*-rhamnoside); KAEMP-*O*-hex—Kaempferol-*O*-hexoside; TFC—total flavonoid content*.

Trials	x_1	x_2	x_3	Peak 10 (MYR-3-*O*-Gal)	Peak 11 (MYR-3-*O*-Rut)	Peak 12 (QUE der)	Peak 13 (QUE-*O*-hex1)	Peak 14 (MYC)	Peak 15 (QUE-*O*-hex2)	Peak 16 (Q-*O*-Galpent)	Peak 17 (QUE-3-*O*-Ara)	Peak 18 (QUE-3-*O*-Rha)	Peak 19 (KAEMP-*O*-hex)	TFC
**1**	**30**	**0.1**	**40**	0.5 ± 0.03	0.6 ± 0.09	1.0 ± 0.19	0.5 ± 0.01	2.0 ± 0.01	0.5 ± 0.06	0.2 ± 0.07	0.8 ± 0.01	1.9 ± 0.14	0.5 ± 0.02	8.6 ± 0.19
**2**	**50**	**0.1**	**40**	0.4 ± 0.07	0.8 ± 0.05	1.7 ± 0.02	0.3 ± 0.04	1.4 ± 0.01	0.8 ± 0.02	0.3 ± 0.03	1.0 ± 0.04	1.5 ± 0.07	0.6 ± 0.03	8.9 ± 0.37
**3**	**30**	**0.2**	**40**	0.4 ± 0.04	0.6 ± 0.03	0.8 ± 0.05	0.3 ± 0.03	1.6 ± 0.37	0.4 ± 0.02	0.2 ± 0.02	0.6 ± 0.05	1.2 ± 0.13	0.4 ± 0.02	6.5 ± 0.75
**4**	**50**	**0.2**	**40**	0.4 ± 0.10	0.6 ± 0.08	0.9 ± 0.12	0.4 ± 0.13	1.6 ± 0.38	0.2 ± 0.06	0.3 ± 0.05	0.9 ± 0.06	1.2 ± 0.32	0.5 ± 0.01	7.1 ± 1.27
**5**	**30**	**0.15**	**30**	0.6 ± 0.12	0.8 ± 0.10	1.2 ± 0.08	0.5 ± 0.14	2.2 ± 0.10	0.6 ± 0.23	0.2 ± 0.10	0.9 ± 0.03	2.0 ± 0.23	0.5 ± 0.01	9.5 ± 1.13
**6**	**50**	**0.15**	**30**	0.5 ± 0.08	0.8 ± 0.19	1.3 ± 0.11	0.5 ± 0.04	2.1 ± 0.02	0.5 ± 0.04	0.3 ± 0.03	1.1 ± 0.07	1.9 ± 0.07	0.6 ± 0.01	9.6 ± 0.67
**7**	**30**	**0.15**	**50**	0.5 ± 0.24	0.6 ± 0.16	1.1 ± 0.11	0.4 ± 0.21	1.9 ± 0.30	0.5 ± 0.01	0.3 ± 0.06	0.8 ± 0.01	1.9 ± 0.02	0.5 ± 0.02	8.5 ± 1.08
**8**	**50**	**0.15**	**50**	0.6 ± 0.11	0.8 ± 0.18	1.3 ± 0.38	0.6 ± 0.10	2.2 ± 0.36	0.6 ± 0.22	0.4 ± 0.05	1.0 ± 0.33	2.0 ± 0.03	0.6 ± 0.13	10.1 ± 0.74
**9**	**40**	**0.1**	**30**	0.4 ± 0.02	0.6 ± 0.02	1.1 ± 0.01	0.3 ± 0.03	2.0 ± 0.15	0.5 ± 0.03	0.2 ± 0.02	0.8 ± 0.03	1.7 ± 0.03	0.5 ± 0.03	8.0 ± 0.02
**10**	**40**	**0.2**	**30**	0.4 ± 0.06	0.7 ± 0.11	1.1 ± 0.10	0.4 ± 0.07	1.6 ± 0.02	0.4 ± 0.05	0.2 ± 0.11	0.7 ± 0.03	1.5 ± 0.13	0.5 ± 0.01	7.5 ± 0.67
**11**	**40**	**0.1**	**50**	0.3 ± 0.06	0.6 ± 0.11	1.1 ± 0.03	0.4 ± 0.04	1.6 ± 0.63	0.1 ± 0.01	0.3 ± 0.05	0.6 ± 0.03	1.4 ± 0.12	0.5 ± 0.03	6.9 ± 1.08
**12**	**40**	**0.2**	**50**	0.3 ± 0.03	0.5 ± 0.02	0.7 ± 0.03	0.3 ± 0.02	1.9 ± 0.57	0.6 ± 0.16	0.3 ± 0.07	0.7 ± 0.04	1.4 ± 0.02	0.3 ± 0.03	7.1 ± 0.30
**13**	**40**	**0.15**	**40**	0.4 ± 0.05	0.6 ± 0.03	0.8 ± 0.13	0.4 ± 0.07	1.4 ± 0.15	0.1 ± 0.03	0.2 ± 0.03	0.5 ± 0.11	1.1 ± 0.13	0.3 ± 0.09	5.8 ± 0.60

* TFC – sum of the amount of each individual flavonoid.

Table S3. Regression coefficients (intercept, linear, quadratic and interaction) of the models obtained for each response (total tannin – TTC, total flavonoids – TFC, myricitrin – MYC, and total polyphenols – TPC – contents).

Model term for each response	Coefficients	Standard error	*p*-value
$TPC = 44.0 + 0.64\ x_1 - 2.26\ x_2 + 0.11\ x_3 - 3.82\ x_1^2 - 2.45\ x_2^2 + 6.03\ x_3^2 + 1.27\ x_1x_2 + 0.41\ x_1x_3 - 0.88\ x_2x_3$			
b0	44.0	1.48	0.000*
b1	0.64	0.90	0.510
b2	−2.26	0.90	0.054
b3	0.11	0.90	0.908
b11	−3.82	1.33	0.035*
b22	−2.45	1.33	0.125
b33	6.03	1.33	0.006*
b12	1.27	1.28	0.365
b13	0.41	1.28	0.763
b23	−0.88	1.28	0.522
$TTC = 34.56 + 0.56\ x_1 - 1.60\ x_2 + 0.43\ x_3 - 4.59\ x_1^2 - 2.14\ x_2^2 + 4.47\ x_3^2 + 0.63\ x_1x_2 - 0.18\ x_1x_3 - 0.87\ x_2x_3$			
b0	34.56	1.21	0.000*
b1	0.56	0.74	0.481
b2	−1.60	0.74	0.083
b3	0.43	0.74	0.580
b11	−4.59	1.09	0.008*
b22	−2.14	1.09	0.108
b33	4.47	1.09	0.009*
b12	0.63	1.05	0.573
b13	−0.18	1.05	0.868
b23	−0.87	1.05	0.446
$TFC = 6.38 + 0.33\ x_1 - 0.51\ x_2 - 0.24\ x_3 + 1.72\ x_1^2 - 0.32\ x_2^2 + 1.34\ x_3^2 + 0.08\ x_1x_2 + 0.39\ x_1x_3 + 0.18\ x_2x_3$			
b0	6.38	0.39	0.000*
b1	0.33	0.24	0.230
b2	−0.51	0.24	0.090
b3	−0.24	0.24	0.363
b11	1.72	0.35	0.005*
b22	−0.32	0.35	0.407
b33	1.34	0.35	0.013*
b12	0.08	0.34	0.817
b13	0.39	0.34	0.298
b23	0.18	0.34	0.610
$MYC = 1.52 - 0.07\ x_1 - 0.05\ x_2 - 0.03\ x_3 + 0.24\ x_1^2 - 0.09\ x_2^2 + 0.37\ x_3^2 + 0.15\ x_1x_2 + 0.09\ x_1x_3 + 0.19\ x_2x_3$			
b0	1.52	0.09	0.000*
b1	−0.07	0.05	0.235
b2	−0.05	0.05	0.394
b3	−0.03	0.05	0.591
b11	0.24	0.08	0.032*
b22	−0.09	0.08	0.300
b33	0.37	0.08	0.007*
b12	0.15	0.08	0.115
b13	0.09	0.08	0.280
b23	0.19	0.08	0.062

The models are presented based on the coded values of the variables. The asterisks (*) indicate the significant coefficients ($p \leq 0.05$).

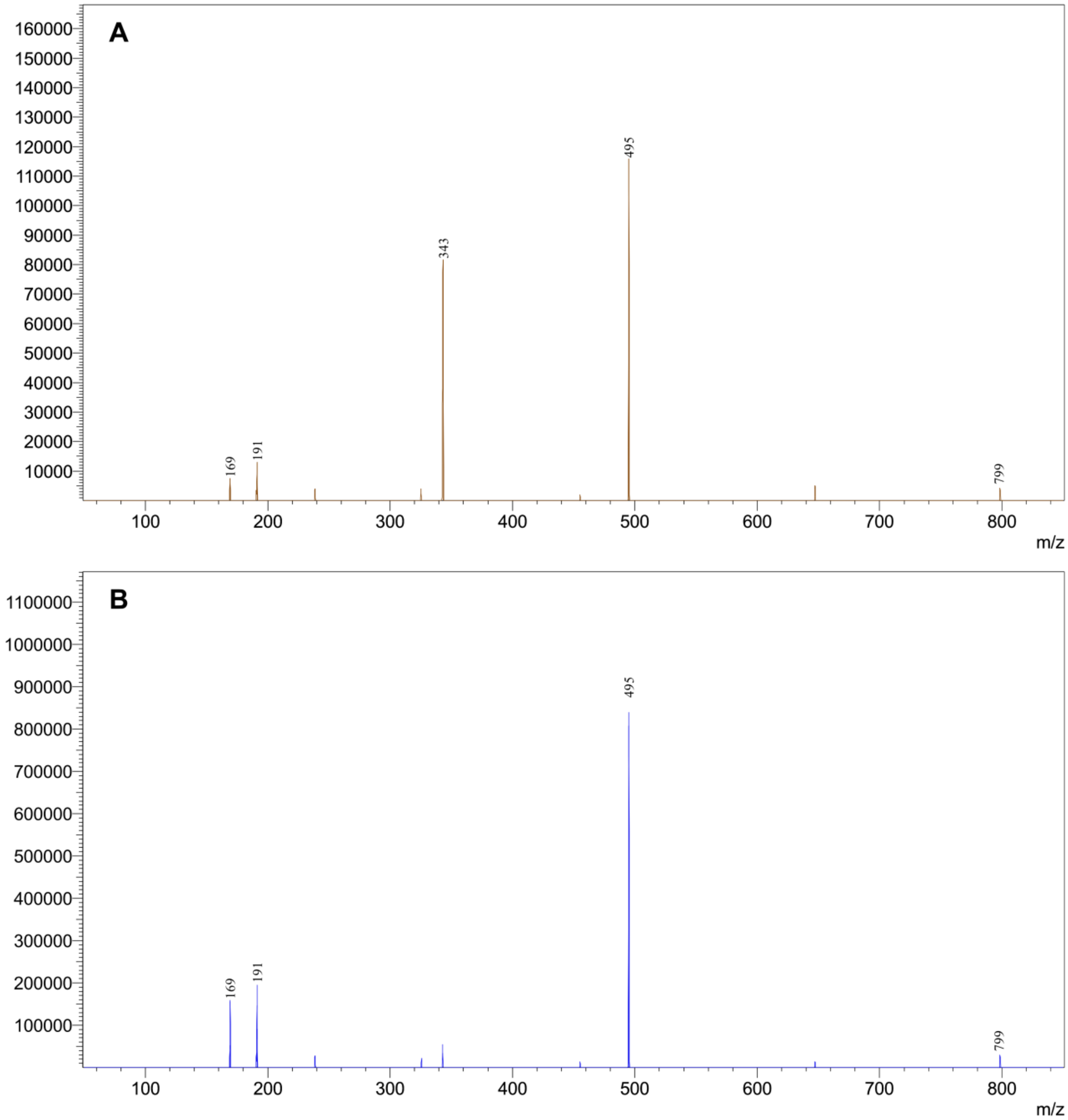

Figure S1. MS/MS spectra of tetragalloyl quinic acids (isomer 1, A and isomer 2, B) corresponding to peak 8 and 9 of Table 4, respectively.
